# An overview of Indian research in obsessive compulsive disorder

**DOI:** 10.4103/0019-5545.69233

**Published:** 2010-01

**Authors:** Y. C. Janardhan Reddy, Naren P. Rao, Sumant Khanna

**Affiliations:** Department of Psychiatry, National Institute of Mental Health and Neurosciences (NIMHANS), Hosur Road, Bangalore - 560 029, Karnataka, India

**Keywords:** OCD, Research, India

## Abstract

Obsessive-compulsive disorder (OCD) was considered a relatively rare disorder until about two decades ago. Since then, considerable advance has been made in understanding the various aspects of OCD that include epidemiology, clinical features, comorbidity, biology and treatment. In the last one decade, there has also been interest in a group of related disorders called obsessive-compulsive spectrum disorders. There is substantial research from India on various aspects of OCD, particularly from the National Institute of Mental Health and Neurosciences (NIMHANS), Bangalore. We attempt to review all the relevant Indian data on OCD.

## ADULT OBSESSIVE-COMPULSIVE DISORDER

### Epidemiology

There is only one epidemiological study from India.[1] The study found lifetime prevalence of 0.6%. This rate is considerably lower compared to the 2-3% rate reported in the European and North American studies.[2,3] However, similar low rate ranging from 0.5-0.9% was observed in a study from Taiwan.[4] It is not clear why lifetime prevalence rate of OCD is lower in some countries although the rates are not very low compared to the conservative estimate of 1% rate of OCD.[5] However, further research is needed into the epidemiological aspects of OCD in India since the data available is limited.

### Phenomenology of obsessive-compulsive disorder in adults

Phenomenology has been an important area of research in the field of OCD that has attracted the attention of Indian researchers. The earliest such study was by Dutta Ray in 1964[6] followed by a series of articles by Akhtar *et al*. on phenomenology and socio-cultural determinants of symptoms in OCD,[7–9] Chakraborty and Banerji, in a study that compared 200 “obsessionals” with 200 controls reported a high rate of family history of obsessional illness (26%) and premorbid obsessional personality (26%).[10] Two other studies also reported high rates of obsessive personality.[11,12]

Khanna *et al*. in an exploratory study examined whether a reactive-endogenous dichotomy exists.[13] Acute onset and fluctuating course was significantly commoner in the reactive subgroup. In an attempt to clarify the nosological status of OCD, Gojer *et al*. compared 53 cases of OCD with an equal number of subjects with depression and anxiety neurosis.[14] There were more similarities in the OCD and anxiety neurosis group than the depressive group.

Khanna and Channabasavanna developed a classificatory system for obsessions and compulsions based on form and content.[15,16] Obsessions were categorized into six categories of form and twelve categories of content and compulsions in to four categories of form and eight categories of content. In the same sample of patients, phenomenology was analyzed using cluster analysis.[17] Four reliable clusters were derived using variables present in 10-90% of the subjects: Washing, checking, thoughts of past and embarrassing behavior. Depression occurred as a unique cluster. Subtypes of OCD were also examined in the same sample.[18] The study showed that washers and checkers are valid subtypes of OCD.

In another study,[19] 222 consecutive subjects were evaluated using the Yale-Brown Obsessive-Compulsive Scale (Y-BOCS) symptom checklist[20] and the Scale for Assessment of Form and Content (SFC).[21] The data was subjected to factor analysis with varimax rotation. The main factors that emerged were washers, checkers, hoarding and two pure obsession factors. The obsession groups had a preponderance of sexual and religious themes. The findings are largely in concordance with those of studies from other parts of the world suggesting similarity across cultures.[22,23] The study, however, supports separating obsessions from compulsions because two pure obsession factors emerged, which is in keeping with the findings of the two previous studies.[24,25] Three recent studies of OCD in adults have also used the Y-BOCS to measure obsessive-compulsive symptoms.[19,26–28] The phenomenology of OCD in these studies is similar to that described in the western population.[29]

Jaisoorya *et al*. examined gender differences in OCD.[30] Males had an early onset of OCD, and had a higher prevalence of symmetry/religious obsessions, miscellaneous compulsions, and comorbid attention deficit hyperactivity disorder (ADHD). Females had higher prevalence of cleaning compulsions and comorbid trichotillomania.

Kamath *et al*. examined suicidal behavior in 100 consecutive DSM-IV OCD patients;[28] 59% had ‘worst ever’ (lifetime) suicidal ideation and 28% had current suicidal ideation. History of suicidal attempt was reported in 27% of the subjects. Major depression, unmarried status and hopelessness were the major risk factors for suicidal behavior.

Gururaj *et al*. assessed the family burden, quality of life and disability in OCD patients and compared them with patients with schizophrenia of comparable severity.[31] Patients with schizophrenia had higher family burden but were comparable to OCD patients with respect to quality of life and disability. The study showed that OCD patients were associated with significant disability, poor quality of life and high family burden comparable to schizophrenia.

### Insight into obsessive-compulsive disorder

Traditionally, OCD is described as a condition in which patients have good insight into their symptoms. The DSM-IV field trial demonstrated a broad range of insight with 30% having poor insight.[32] Subsequent studies have also reported poor insight in 15-36% of patients with OCD.[33–36] The DSM-IV has added a new OCD specifier: “With poor insight” which involves a lack of recognition that the symptoms are unreasonable or excessive.[37]

There is paucity of data regarding the clinical correlates and treatment response of poor insight in OCD. A significant limitation of most of the studies is that they did not use validated measure of insight. Only one study[33] used the Brown Assessment of Beliefs Scale (BABS) developed specifically to assess insight.[38] In a recent Indian study, demographic and clinical correlates of poor insight OCD, and the association between response to specific serotonin reuptake inhibitors (SSRIs) and baseline insight was examined in a sample of 100 DSM-IV OCD subjects by using the BABS as a measure of insight.[27] The sample had 25 subjects with poor insight and the remaining 75 had good insight. Those with poor insight had earlier age-at-onset, more severe illness, higher comorbidity rate particularly major depression, over representation of miscellaneous obsessions and hoarding and poorer treatment response. The study suggests that OCD with poor insight could be a distinct subtype. That a significant proportion of OCD patients have poor insight has important treatment implications. Patients with poor insight could easily get misdiagnosed as psychotic and treated accordingly. The study suggests that drug treatment response is poor in those with poor insight. The finding is in sharp contrast to the findings of a previous study that reported that degree of insight at baseline did not predict response to sertraline.[33] It is clinically pertinent to examine if poor insight patients do better with addition of neuroleptics. There is, however, no evidence as yet to suggest that those with poor insight respond better to augmentation with antipsychotics. On the other hand, a few studies have shown that insight improves after treatment with SSRIs.[35,36]

### Comorbidity

Studies of comorbidity are varied and have examined a broad range of topics including spectrum disorders, comorbidity with schizophrenia and bipolar disorder and even prevalence of OCD in Parkinson’s disease.

Obsessive-compulsive (OC) spectrum disorders have over the past few years emerged as a unique and fascinating category of related conditions.[39] Jaisoorya *et al*. examined the prevalence of putative OC spectrum disorders in a large sample of OCD subjects (n = 231) in comparison with relatives of neurologically ill patients (n = 200).[26] Prevalence of tic disorders (39% vs. 12%), hypochondriasis (13% vs. 0), BDD (3% vs. 0) and trichotillomania (3% vs. 0) were significantly greater in OCD subjects compared to controls. However, the prevalence of sexual compulsions, pathological gambling, eating disorders, and depersonalization disorder was not greater in the OCD subjects compared to controls. The findings of this study suggest that tic disorders, hypochondriasis, BDD, and trichotillomania are perhaps part of the OC spectrum disorders. The evidence for exclusion of other disorders from the hypothesized OC spectrum is not conclusive because of the rarity of the occurrence of some of these disorders in the study sample. The findings are somewhat similar to those of a study that reported high rates of BDD, hypochondriasis and low rate of eating disorders and most impulse control disorders other than pathological skin picking.[40] Only one patient in the sample had an eating disorder. The finding is in sharp contrast to a previous study that reported high rates of eating disorders among OCD patients.[41]

This divergence should be viewed in the light of the rare reporting of eating disorders in Asian countries[42,43] but could well be a correlate of cultural beliefs and attitudes that have been identified as significant contributing factors in the development of eating disorders.[43] Jaisoorya *et al*. examined the differences between tic related and non tic related OCD with respect to sociodemographics, symptom profile, and comorbidity.[44] Tic related OCD had an early age at onset, over representation of males, aggressive obsessions, cleaning compulsions and comorbid trichotillomania.

In a chart review of comorbidity in 218 OCD subjects, 17% had major depression, 6% dysthymia, and 7% any anxiety disorder.[45] Comorbidity rates were low and there were not many differences between those with and without comorbidity except that female subjects were more likely to have depression. Kalra *et al*. compared OCD with and without comorbid Axis I disorders in a sample of 54 subjects and found that those with comorbidity had higher scores on depression and OCD severity scales.[46] The study findings were in tune with earlier literature from rest of the world. Gupta *et al*. examined level of comorbid depression in patients with OCD, psychotic depression and chronic medical illness.[47] All three groups had moderate to high levels of depression, with OCD group intermediary between psychotic depression and physical illness. However, the OCD group had more life events than depression or physical illness.

Rajkumar *et al*.[48] studied the clinical profile of schizophrenic patients with and without comorbid OCD (50 in each group). Schizo-obsessive patients had higher rates of paranoid symptoms and first-rank symptoms of schizophrenia. They had lower anergia, higher depression scores, more comorbid personality disorders, and disability. Significant correlations were observed between OCD severity scores and schizophrenia symptom dimension scores. Authors concluded that “schizo-obsessive” schizophrenia may be a distinct subtype with unique clinical characteristics.

A retrospective chart analysis of 15 cases OCD with psychosis found that obsessive doubts, washing and checking compulsions were the most common OC symptoms.[49] Twelve cases had a diagnosis of schizophrenia, while three had atypical psychosis. About half the patients had First Rank symptoms of schizophrenia. Nearly three-fourth of the sample showed significant improvement on treatment with a combination of antipsychotic and antiobsessional drugs.

Zutshi *et al*.[50] examined differences between bipolar OCD and non-bipolar OCD. Bipolar OCD was associated with episodic course, a higher family loading for mood disorders, and higher rates of comorbid depression, social phobia and generalized anxiety disorder. In majority of the patients, OCD predated bipolar disorder and OCD worsened during depression and improved during mania. Authors concluded that OCD in those with bipolar disorder may be pathophysiologically related to bipolar disorder.

Harbishettar *et al*.[51] systematically assessed OC symptoms and OCD in 69 Parkinson’s disease patients and matched medically ill controls. There was no difference between the groups with respect to OC symptoms, OCD both clinical and subclinical and tics. Also, there was no relationship between severity of Parkinson’s disease and OC symptoms. Authors speculated that different circuitry may be involved in the pathophysiology of OCD and Parkinson’s disease although basal ganglia involvement may be common to both the disorders.

### Course and outcome

There is limited literature on the long-term course and outcome of OCD. In an 11-13 year follow-up study of 75 subjects with OCD, Reddy *et al*.[52] reported a favorable outcome in majority of the subjects: 43% had no OCD, 33% had subclinical OCD and only 24% had clinical OCD. Median time to reach ‘no OCD’ and ‘subclinical’ status was 42 months and 84 months respectively. Interestingly, 37% were in true remission (‘no OCD’ and not on any treatment) for a median period of 132 months. Those who had ‘mixed’ OCD and Axis I comorbidity had poorer outcome. Age of onset and duration of illness had no effect on outcome. Optimistic outcome reported in this study is somewhat different from the findings of studies from other parts of the world which have reported lower rates of remission. Previous studies included samples that were severe and chronically ill with high rates of comorbidity. The subjects in the study by Reddy *et al*. were largely self-referred, moderately ill, and did not have history of treatment resistance. The findings of this study, therefore, could be generalized for patients routinely seen in the outpatient consultation at clinics and secondary-care hospitals in India.

Math *et al*.[53] in another follow-up study explored if the long term outcome of ‘predominantly obsessive’ subjects differs from that of ‘mixed’ OCD. They studied the five to six-year course and outcome of 54 patients with ‘predominantly obsessions’ and 54 with ‘mixed’ subtype of OCD. The course of the illness was similar in both and a majority (72%) did not have clinical OCD at follow up.

In another study, Shetti *et al*.[54] examined the differences between SSRI responders and non responders. They assessed 67 SRI responders and 55 non responders. Base line severity of illness, comorbid major depression, sexual obsessions, washing and miscellaneous compulsions, early age at onset, ‘mixed’ OCD and poor insight were associated with poor response to SRIs.

## NEUROBIOLOGY

### Neurotransmitters in obsessive-compulsive disorder

A serotonergic hypothesis of OCD was suggested originally by the observed differential efficacy of SRIs in alleviating OCD symptoms.[55] Since then, numerous studies of peripheral receptor binding in the blood or concentrations of serotonin metabolites in cerebrospinal fluid have been performed but have yielded inconsistent results.[56] Pharmacological challenge studies provide another indirect approach. By administering serotonergic agents and measuring endocrine and behavioral responses, investigators have attempted to study the central serotonergic functioning in OCD. It is observed that OCD patients become significantly more anxious and dysphoric after administration of meta-chlorphenyl-piperazine (mCPP), a 5-HT receptor agonist.[55] In addition, obsessive-compulsive symptoms worsen. However, there appears to be blunted cortisol and prolactin response in response to mCPP. In an attempt to replicate these findings, mCPP was administered orally in a randomized double-blind design to 34 OCD patients who were either drug-naïve or drug-free for a minimum of four weeks.[57] The cortisol and prolactin responses were contrasted with those of 18 drug-free healthy subjects. The OCD patients showed significantly blunted cortisol and prolactin responses to mCPP challenge as compared to normal subjects. However, mCPP did not produce any significant exacerbation of obsessive-compulsive symptoms in the patients. These findings are suggestive of a serotonin (5-HT) receptor hyporesponsivity in the HPA axis. Even though previous studies indicate a hyperresponsivity of the 5-HT receptor system as shown by significant symptom worsening following serotonergic challenge,[58,59] the Indian study failed to replicate those results.[58] It was postulated that the 5-HT receptor hyporesponsivity in the HPA axis may be a biological “trait marker” of OCD, and may not be directly involved in the mediation of symptomatology of the disorder. It could also be inferred that the discrepancy among studies regarding the behavioural responses to mCPP challenge may in part be due to differences in the basic environmental conditions across various studies.[60] In a previous study by the same group, an endocrinological blunting in the absence of a behavioural increase in obsessive-compulsive symptoms was documented after oral administration of mCPP; however, when exposure was incorporated into the paradigm, with oral mCPP, exacerbation of obsessive-compulsive symptoms was noted.[61]

A normal endocrinological response after treatment with clomipramine was also independently documented.[62] It is a matter of conjecture whether stimulation of noradrenergic system by the α2 adrenergic antagonistic action of mCPP, or behavioral exposure conditions during the challenge procedure are also partly responsible for the symptom exacerbation as noted in previous studies.[57]

In summary, pharmacological challenge studies and other studies that have explored serotonergic hypothesis in OCD, have very limited evidence to support a primary serotonergic dysfunction in OCD.[63] However, a modulation of serotonergic system clearly plays a role in effective pharmacotherapy of at least a significant proportion of OCD patients.

In a study by Khanna *et al*. there was a blunted growth hormone, cortisol and ACTH response to clonidine in OCD.[64] On qualitative analysis three possible responses of growth hormone were obtained: Accentuation (>10 ng/ ml), normal (5-10 ng/ml) and attenuation (<10 ng/ml). Most patients with an accentuated response were patients with compulsions, pure obsessions were significantly more likely to have blunted responses. The study findings suggest noradrenergic dysfunction in OCD and also imply noradrenergic heterogeneity in the observation that pure obsessions tend to have a more down regulated noradrenergic system as compared to the compulsives. Based on their work, Khanna *et al*. concluded that serotonergic hypothesis may not explain all the abnormalities seen in OCD and that complex interactions between various neurotransmitters as well as the environmental conditions may be necessary to cause OCD.[57]

### Soft neurological signs

Thirty-seven drug free non-depressed OCD subjects and 20 normal healthy volunteers were screened for SNS.[65] The OCD subjects had significantly more total SNS as compared to normals. These findings were most marked in the frontal lobe functions. There was a trend towards significance in temporal lobe functions, while other test findings were not impaired. If individual items were studied the problems were predominantly in complex motor tasks. There was no significant laterality effect.

### Electrophysiological studies

Most electrophysiological studies in OCD have either tried to localize the site of the disorder at a structural or functional substrate, or have been based on the associated increased autonomic arousal. Khanna concluded that in most cases there was no obvious EEG abnormality in OCD; when it was present it was likely to be a non-specific disturbance in the temporal and frontotemporal regions.[66]

In OCD there was a decreased power in the nondominant frontomedial and posterior temporal regions in the computerized EEG analysis. There were no significant differences in the coherence observed from these sites.[67] The study suggested nondominant frontomedial hypofunctioning to be associated with OCD.

In a study of resting middle latency auditory and visual evoked potentials in 50 OCD subjects and 40 normal controls, there were no significant differences between the two groups for amplitude and latency or left-right ratios.[68] The study did not support any laterality deficit in OCD and was inconsistent with the hypothesis of left frontal lobe dysfunction in OCD.[69] A more prolonged post imperative negativity and a higher amplitude of the late component of the Contingent Negative Variation (CNV) has been repeatedly recorded.[66] OCD patients exhibited higher amplitude of the ‘late’ component of the CNV. The role of the mesencephalic reticular formation with modulation by the frontal granular cortex in the genesis of these potentials has been stressed.

Bereitschafts potential has been found absent or to have a decreased onset latency in 44 subjects with OCD.[70] A deficit of the complex motor programming circuit similar to those observed in Gilles de la Tourette syndrome has been put forth on the basis of this observation.[70] Based on the evidence from electrophysiological, neuropsychological, scan, lesion, and psychosurgical studies, Khanna also proposed an integrated model of possible frontal dysfunction in OCD with associated dysfunction in other areas of the brain such as cingulum and basal ganglia.[66]

### Immunological factors

Khanna *et al*. documented increased levels of serum immunoglobulins in OCD subjects as compared to normal controls, with specific reference to IgG.[71] The IgG levels were high even after clinical improvement. The authors speculated that the immunological abnormality could be a marker of vulnerability to OCD. They also discussed the possibility that the immunological dysfunction could be due to an unidentified infectious agent or an autoimmune process. As an extension of the hypothesis, viral antibodies were measured in the blood[72] and cerebrospinal fluid (CSF) of OCD subjects.[73] IgG viral antibodies for herpes simplex virus-1 (HSV-1), varicella zoster, cytomegalo virus, measles and mumps were studied in 76 subjects with OCD and compared with 55 normal healthy volunteers. There was a significantly higher titer for HSV-1 antibodies in both serum and CSF. The sera: CSF ratios were suggestive of intrathecal synthesis. The study on viral antibodies in CSF suggests a role for HSV-1 in OCD. However, caution needs to be exercised in interpreting the finding because of certain methodological issues raised in the paper by the authors.

Exploration of the contribution of immunological mechanisms in the manifestation of OCD continued in a recent study by Bhattacharya *et al*. that investigated the presence of auto antibodies directed against the basal ganglia or thalamus in the serum as well as CSF of 23 OCD patients compared with 23 matched psychiatrically normal controls using western blot.[74] They further investigated CSF amino acid (glutamate, GABA, taurine, and glycine) levels and examined the extent to which these levels were related to the presence of auto - antiibodies. There was evidence of significantly more binding of CSF auto - antiibodies to homogenate of basal ganglia as well as to homogenate of thalamus among OCD patients compared to controls. There was no significant difference in the pattern of binding between patients and controls using serum. CSF glutamate and glycine levels were also significantly higher in OCD patients compared with controls, and CSF glycine levels were also significantly higher in those OCD patients who had auto - antiibodies compared to those without. The study implicates autoimmune mechanisms in the pathogenesis of OCD and also provides preliminary evidence that auto antobodies against basal ganglia and thalamus may cause OCD by modulating excitatory neurotransmission.

In support of the possible immunological mechanisms in the causation of at least some forms of OCD, a few clinical studies have examined the association between infections and OCD. A study reported OCD in some cases of Herpes Simplex encephalitis.[75] In a study of 20 subjects with rheumatic chorea, four subjects (20%) had OCD.[76] The relationship between OCD and rheumatic chorea and Pediatric, Autoimmune, Neuropsychiatric Disorders Associated with Streptococcal infections (PANDAS) is well known.[77] Considering the association between rheumatic fever and OCD, and possible long term neuropsychiatric sequelae in those with history of rheumatic fever because of possible autoimmune insult to basal ganglia, a study recently examined the prevalence of OCD in adults with Rheumatic Heart Disease (RHD).[78] Of the 100 subjects with RHD, 10 had clinical OCD. This rate is at least five-fold higher than the reported global general population rate of OCD[5] and over 15-fold higher than the 0.6% rate of OCD in India.[1] The finding lends support to the hypothesis that OCD could be a long term complication of autoimmune basal ganglia insult in childhood just as RHD is a long term sequel of autoimmune damage to the heart. The results of this study need to be replicated in a controlled study.

Chakrabarty *et al*. investigated glutamatergic dysfunction linked to immune pathogenesis in 21 OCD patients and 18 healthy controls by collecting CSF.[79] They estimated glutamate levels and found that OCD patients had higher glutamate levels. Age, gender, duration of illness, severity of illness did not have any effect on glutamate levels.

## NEUROPSYCHOLOGY

Neuropsychological studies have provided important clues in understanding the neurobiological basis of OCD. As neuropsychological deficits are potential endophenotype markers, studies have examined patients in symptomatic phase, recovered phase and also in unaffected first degree relatives.

Trivedi *et al*.[80] examined executive functions, vigilance and spatial working memory in 30 OCD patients and 30 age and education matched control subjects. OCD patients had significant deficits in all the cognitive domains. There was a positive correlation between severity of illness and attention deficits but there was no correlation between duration of illness and cognitive dysfunction. A study by Tarafder *et al*.[81] examined neuropsychological disposition and executive functions in 20 OCD patients and 20 matched normal healthy controls. Subcortical-cerebellar-spinal domain was found to be associated with cognitive style and executive functions, affirming the neurobiological basis of the disorder.

Rao *et al*. examined neuropsychological deficits in 30 recovered OCD patients in comparison with 30 matched healthy controls.[81] They were assessed on tasks for attention, executive function, memory and intelligence. Patients had significant deficits in tests of set shifting ability, alternation, response inhibition and non verbal memory. There was no correlation between illness related variables neuropsychological deficits. The study findings suggest neuropsychological deficits are possibly state independent.

In a recent study by Viswanath *et al*. 25 unaffected siblings of probands with familial OCD in comparison with 25 matched healthy controls had significant deficits in tests of decision making and behavioral reversal but not in other tests of attention, executive function, intelligence and memory.[82] The deficits are consistent with the proposed neurobiological model of OCD involving the orbitofrontal cortex and suggest that the deficits could be potential endophenotypes in OCD.

### Family studies

Methodologically sound studies in the last decade have reported higher morbid risk for OCD among first-degree relatives of OCD probands[83,84] but Indian studies have reported either no increase in morbid risk[85] or much less than what was previously reported.[86] The rate of OCD in 135 first-degree relatives of 33 adult OCD probands was comparable to the rate in 148 adults from the general population in the study by Guruswamy *et al*.[85]

In the family study of juvenile OCD, that examined first-degree relatives of 35 juvenile OCD probands and 34 matched normal controls,[86] the morbid risk for OCD among relatives of OCD probands was 5%, while none of the relatives of controls had OCD. In addition, none of the relatives had Tourette syndrome and only one relative of OCD proband had chronic tics. The study concluded that most juvenile cases of OCD were nonfamilial and unrelated to tic disorders, while only a few were familial.

Sagnik *et al*. examined familiality of washers and checkers by interviewing first-degree relatives of 25 checkers, 30 washers and 40 psychiatrically normal control probands.[87] The morbid risk of OCD was significantly higher among relatives of checker probands (19.4%) than in the relatives of washer (8.7%) or control probands (5.4%), while the morbid risk for relatives of washer and control probands was not significantly different. In all, 67% of the checker relatives with OCD were checkers, while 54% of the washer relatives with OCD were washers. The study provided preliminary evidence of familiality of the checker subtype of OCD.

### Miscellaneous

Chakraborty *et al*. examined the role of oxidative stress in pathogenesis of OCD.[88] They estimated serum Thiobarbituric Acid Reacting Substances (TBARS) formed as a result of free radical lipid peroxidation in 39 newly diagnosed drug free OCD patients and 33 disease free control subjects. Patients had significantly higher TBARS than controls. In addition, there was a strong positive correlation between TBARS and the disease severity. The study suggests that oxidative stress induced increased free radical are generated in OCD patients.

## OCD IN CHILDREN AND ADOLESCENTS

### Demographics

In all the studies of OCD in children and adolescents reported from India, males have outnumbered female subjects.[86,89–91] Male preponderance in juvenile OCD is consistent with the previous clinical studies of juvenile OCD justifying the argument that gender distribution in OCD is developmentally sensitive.[92]

### Phenomenology

A study by Khanna and Srinath from India was one of the earliest studies to systematically examine the clinical profile of OCD in children in comparison with the OCD in adults.[93] In this sample, obsessions were less frequent compared to compulsions. Obsessions of harm, religion, and impersonal images were commonly reported. Washing, praying, touching, counting and spitting were the common compulsions.

Recent studies from India[89,90] have examined the phenomenology of OCD in children using the chilldren’s version of the Yale-Brown Obsessive-Compulsive Scale (CY-BOCS), the instrument that is widely used all over the world.[94] In a study of 58 children and adolescents, all aged 16 years and below,[90] contamination obsessions were the commonest (62%), followed by obsessions related to aggression (57%), symmetry (34%), sex (22%), religion (22%), somatic (12%), and hoarding (7%). Regarding compulsions, cleaning and washing was the commonest (69%) followed by repeating (52%), checking (47%), ordering (29%), counting (15%), and hoarding (7%). The miscellaneous obsessions and compulsions were present in 65% and 47% of the subjects respectively. The phenomenology of OCD in these studies is similar to that reported in a group of 70 young patients at the National Institute of Mental Health (NIMH) in USA.[95]

In one study,[91] the phenomenology in juvenile OCD was compared with that of adult-onset OCD and juvenile-onset adult OCD, in view of the previously reported findings that juvenile OCD could be phenotypically different from adult OCD[96,97] and juvenile-onset adult OCD.[98] Obsessions related to contamination and compulsions related to checking and miscellaneous types were common in juvenile OCD. In addition, the mean Y-BOCS score was greater in the juvenile OCD and juvenile-onset adult OCD subjects compared to the adult-onset OCD subjects suggesting greater severity of OCD in the juvenile groups. The variations in the clinical manifestations support developmental variability in the expression of OCD. However, they are not consistent with specific variations reported in previous studies.[97,98] For example, OCD in juveniles was associated with a higher frequency of aggression/catastrophic obsessions, hoarding and saving compulsions.[97] Sexual obsessions were selectively more prevalent in adolescents compared with children or adults. It is possible that sexual and aggressive obsessions were underrepresented in this sample due to the fact that the subjects kept them secret because of embarrassment and possible guilt associated in revealing them. However, there could also be a true cross-cultural variation in the phenotypic manifestation of OCD.

### Comorbidity

Psychiatric comorbidity is common in adults with OCD. Similarly, studies of juvenile OCD have found high rates of comorbid major depression (10%-73%), anxiety disorders (26%-76%), and tic disorders (17%-59%).[89] Three Indian studies have systematically examined the comorbidity in juveniles with OCD.[89–91] Rates of comorbid major depression, dysthymia, and bipolar disorder have ranged from 14-23%, 0-2%, and 0-2% respectively. Among anxiety disorders, rates of panic disorder, social phobia, specific phobias, overanxious disorder and separation anxiety disorder ranged from 0-6%, 0-13%, 5-7%, 0-7%, and 5-7% respectively.

Of considerable interest is the comorbid relationship between tic disorders, disruptive behavior disorders and juvenile OCD. Rates of TS have varied from 11-15% and that of other tic disorders from 17-59%.[89] In the three Indian studies, rates of TS and chronic tics are in the range of 8-11% and 2-23% respectively. In the follow-up study by Leonard *et al*. TS was present in 15% of the sample and any tics in 59% of the sample.[99] The rate of TS in the Indian juvenile OCD samples is somewhat comparable to the rates in previous studies, but the overall rate of tic disorders and, in particular, chronic tics are somewhat lower. In a recent study, the clinical profile of OCD+ tics patients was examined in juvenile OCD, juvenile-onset adult OCD and adult-onset OCD subjects.[91] Miscellaneous compulsions such as touching, tapping, rubbing, blinking, staring etc (73% vs. 45% vs. 32%) and pathological doubts (40% vs. 13% vs. 9%) and ADHD (26% vs. 3% vs. 0) were over represented in the juvenile OCD group compared to the other two groups. The miscellaneous compulsions of the type reported in this study were also reported in previous studies of OCD patients with tics[100,101] but the obsessions are not similar to the ones reported in other studies that found mainly excess of aggressive, sexual, and symmetry obsessions.[101,102] Further, the elevated rate of ADHD in juvenile OCD with tics support the previous observations that ADHD, tics and OCD commonly co-occur in juvenile OCD[99,103] and are possibly interrelated sharing a common pathophysiology.[104]

Comorbid ADHD is considered by some to be a developmental marker of juvenile OCD.[105] In the study by Leonard *et al*.[106] the rate of ADHD was 26% and in the studies by Geller and colleagues,[97,105,107] the rate of ADHD was as high as 57%. In the three Indian studies, rates of ADHD ranged from 3 to 18%.[89–91] The rates of ADHD in Indian samples are considerably lower than the rates reported in previous studies. The samples in the previous studies by Geller and colleagues were recruited from a specialized pediatric OCD program, whereas the Indian samples were largely “self-referred” and this difference in the ascertainment method might possibly explain the variation in the rates across the samples. However, at least in one study,[91] the 18% rate of ADHD was higher than the 5-10% rate reported in community samples.[108,109] The elevated rate of ADHD in juvenile OCD in this study is consistent with the findings of previous studies[97,105,107] although the rate of ADHD is much lower than the 51-57% in children and 36-39% in adolescents reported in the studies by Geller and others.[97,107]

In the study by Jaisoorya *et al*. juvenile OCD was compared with adult-onset OCD, using multinomial logistic regression analysis.[91] There was positive association of chronic tics, ADHD, major depressive disorder, and Body Dysmorphic Disorder (BDD) with juvenile OCD. The TS showed an almost significant association with juvenile OCD. The BDD also had a positive association with juvenile-onset adult OCD. In addition regression analysis (juvenile-onset adult OCD vs. adult-onset OCD), showed positive association between social phobia, chronic tics and MDD and juvenile-onset adult OCD. These findings suggest that there are age-specific correlates of the disorder across the life cycle. Further, the findings suggest that OCD in juveniles is perhaps a developmental subtype of OCD with specific correlates such as high rate of ADHD and tic disorders.[96,97,107]

## COURSE AND OUTCOME OF JUVENILE OBSESSIVE-COMPULSIVE DISORDER

Follow-up studies of OCD in children and adolescents have reported low rates of remission.[106,110–112] Similarly, studies of adult OCD have reported worse course in those with early onset of illness.[113,114] However, studies on long-term course and outcome of OCD in juveniles are few and many have small sample sizes. We discuss here a two to nine year follow-up study of 58 children and adolescents with DSM-III-R OCD from India.[90] The subjects were largely ‘self-referred’ (93%) and ‘drug-naïve’ (90%) at the time of consultation. None had received any form of psychotherapeutic intervention and none were treatment refractory at the time of first consultation. Most were treated with medications and only a few of them with a combination of medicines and exposure and response prevention. At the time of follow-up, only 29% were still receiving medication. The median duration without any treatment at the time of follow-up was 49 months. At follow-up, 62% of the subjects were in full remission or had ‘no OCD’ (Total Y-BOCS score = 0 to 3), 17% had subclinical OCD (Y-BOCS score, 4-15) and only 21% had clinical OCD (Y-BOCS>15). The median time to achieve full remission was 24 months and subjects were symptom free for a mean of 41 months prior to follow-up assessment. However, the most significant finding is that 28 subjects (48%) were in true remission (full remission and not on any treatment) and were not receiving treatment for a mean period of 58 months. Duration of follow-up and age-at-onset emerged as significant predictors of full remission. The odds of younger subjects having full remission or no OCD outcome were 1.5 times that of older subjects. Those who had ‘no OCD’ at follow-up had earlier age-at-onset of illness.

The high rate of ‘true remitters’ is in sharp contrast to the 6% rate in the study by Leonard and others.[106] The rate of clinical OCD (21%) at follow-up is low compared to the high rates of clinical OCD (35%-68%) reported in previous studies.[106,110–112,115] Favorable prognosis in this study could be due to several reasons. First, the sample was largely ‘self-referred’, ‘drug-naïve’, moderately ill, with relatively low rate of comorbidity (55%). In the classic study by Leonard *et al*., the subjects were severely and chronically ill with history of treatment resistance in 75% of them and 100% comorbidity.[106] Second, a low rate of tic disorders (16%) and ADHD (3%) could have contributed to better prognosis.

The study findings suggest that juvenile OCD, at least, in self-referred, drug-naïve outpatient clinical samples has a good prognosis. The findings can be generalized to psychiatric hospital settings in India and perhaps to general psychiatric practice settings in the Western countries.

## SUMMARY

Indian research on various aspects of OCD has shown broad similarities with that of research from the other parts of the world. Clinical profile of OCD seems to be similar to what is described in the literature. Comorbid patterns also appear to be similar across cultures. Follow-up studies have shown that prognosis is favorable in the long-run. There is evidence from a large Indian study that tic disorders, hypochondriasis, BDD and trichotillomania are perhaps part of the putative OC spectrum disorders. However, eating disorders are uncommon in patients with OCD.

Biological research in OCD in India has paralleled the interest in the area in other parts of the world. There seems to be a consensus that serotonergic hypothesis may not explain all the abnormalities in OCD and that complex interactions between various neurotransmitters as well as environmental factors may be necessary to cause OCD. Although not a single case of PANDAS has been reported from India, several studies have shown the possible role of immunological factors in the causation of OCD.

Substantial research has been carried out in juveniles with OCD. The rates of ADHD and TS are somewhat lower in Indian samples compared to those from other parts of the world. There is a suggestion that juvenile OCD could be a developmental subtype of the disorder. Juvenile OCD seems to have a favorable prognosis.

There is surprisingly limited amount of data from India on treatment aspects of OCD. Currently, at NIMHANS, Bangalore there is ongoing research on various aspects of OCD such as clinical profile, course, biology and treatment.
